# A pseudo-outbreak of *Cyberlindnera fabianii* funguria: Implication from whole genome sequencing assay

**DOI:** 10.3389/fcimb.2023.1130645

**Published:** 2023-03-07

**Authors:** Xin Fan, Rong-Chen Dai, Timothy Kudinha, Li Gu

**Affiliations:** ^1^ Department of Infectious Diseases and Clinical Microbiology, Beijing Institute of Respiratory Medicine and Beijing Chao-Yang Hospital, Capital Medical University, Beijing, China; ^2^ School of Public Health, Zhejiang Chinese Medical University, Hangzhou, Zhejiang, China; ^3^ School of Dentistry and Medical Sciences, Charles Sturt University, Leeds Parade, Oranges, NSW, Australia; ^4^ NSW Health Pathology, Regional and Rural, Orange hospital, Orange, NSW, Australia

**Keywords:** uncommon fungal pathogen, molecular typing, nosocomial transmission, *Cyberlindnera fabianii*, whole genome sequencing (WGS)

## Abstract

**Background:**

Although the yeast *Cyberlindnera fabianii* (*C. fabianii*) has been rarely reported in human infections, nosocomial outbreaks caused by this organism have been documented. Here we report a pseudo-outbreak of *C. fabianii* in a urology department of a Chinese hospital over a two-week period.

**Methods:**

Three patients were admitted to the urology department of a tertiary teaching hospital in Beijing, China, from Nov to Dec 2018, for different medical intervention demands. During the period Nov 28 to Dec 5, funguria occurred in these three patients, and two of them had positive urine cultures multiple times. Sequencing of rDNA internal transcribed spacer (ITS) region and MALDI-TOF MS were applied for strain identification. Further, sequencing of rDNA non-transcribed spacer (NTS) region and whole genome sequencing approaches were used for outbreak investigation purpose.

**Results:**

All the cultured yeast strains were identified as *C. fabianii* by sequencing of ITS region, and were 100% identical to the *C. fabianii* type strain CBS 5640T. However, the MALDI-TOF MS system failed to correctly identify this yeast pathogen. Moreover, isolates from these three clustered cases shared 99.91%-100% identical NTS region sequences, which could not rule out the possibility of an outbreak. However, whole genome sequencing results revealed that only two of the *C. fabianii* cases were genetically-related with a pairwise SNP of 192 nt, whilst the third case had over 26,000 SNPs on its genome, suggesting a different origin. Furthermore, the genomes of the first three case strains were phylogenetically even more diverged when compared to a *C. fabianii* strain identified from another patient, who was admitted to a general surgical department of the same hospital 7 months later. One of the first three patients eventually passed away due to poor general conditions, one was asymptomatic, and other clinically improved.

**Conclusion:**

In conclusion, nosocomial outbreaks caused by emerging and uncommon fungal species are increasingly being reported, hence awareness must be raised. Genotyping with commonly used universal gene targets may have limited discriminatory power in tracing the sources of infection for these organisms, requiring use of whole genome sequencing to confirm outbreak events.

## Introduction

Emerging fungal infections have become a global health concern in the past few decades due to their notable morbidity and mortality, especially among immunosuppressed patients admitted to intensive care units (ICUs), or undergoing invasive medical interventions (Pappas et al., 2018; Hoenigl et al., 2022; World Health Organization, 2022). Although *Candida albicans* remains the most predominant yeast pathogen, the incidence of uncommon yeast species causing human infections has increased enormously in recent years (Pappas et al., 2018; Chen et al., 2021). Uncommon yeast species often exhibit decreased susceptibility to commonly used antifungal agents, making them difficult to manage in clinical settings. Moreover, there are increasing incidences of nosocomial infections and outbreak events reported due to transmission of uncommon or emerging yeast species worldwide (Pappas et al., 2018; Chen et al., 2021). For instance, *Candida auris*, which was first described in 2009, has caused a number of outbreaks in different continents (Chow et al., 2018; Hoenigl et al., 2022; World Health Organization, 2022).

In the investigations and tracing of fungal nosocomial transmissions, molecular genotyping could provide essential genetic evidence. Hence, a wide variety of molecular typing assays have been evaluated and implemented in the study of outbreaks, including band-pattern-based DNA analysis like random amplified polymorphic DNA (RAPD) and pulsed field gel electrophoresis (PFGE), traditional DNA sequencing-based phylogenetic methods like single gene analysis, microsatellite analysis and multilocus sequence typing (MLST), protein spectrum analysis by matrix-assisted laser desorption/ionization-time of flight mass spectrometry (MALDI-TOF MS), and whole genome sequencing (WGS) techniques (Reiss et al., 1998; Pulcrano et al., 2012; Mikosz et al., 2014; Xiao et al., 2014; Litvintseva et al., 2015; Oliveira and Azevedo, 2022). Of these methods, WGS has become increasingly used due to its outstanding discriminatory power (Litvintseva et al., 2015; Bougnoux et al., 2018; Desnos-Ollivier et al., 2020; Oliveira and Azevedo, 2022).

In this study, we report on three clustered funguria cases caused by a rare fungal pathogen, *Cyberlindnera fabianii*, which occurred over a two-week period within the same urology department, which was initially considered as a nosocomial outbreak event. Using WGS, this event was finally confirmed as a pseudo-outbreak caused by *C. fabianii* from two diverged genetic lineages.

## Material and methods

### Ethics

This study was approved by the Human Research Ethics Committee of the Beijing Chaoyang Hospital (No. KE332). Written informed consent was obtained from all participants involved.

### Routine isolation of the microorganisms and MALDI-TOF MS identification.

**Figure 1:**
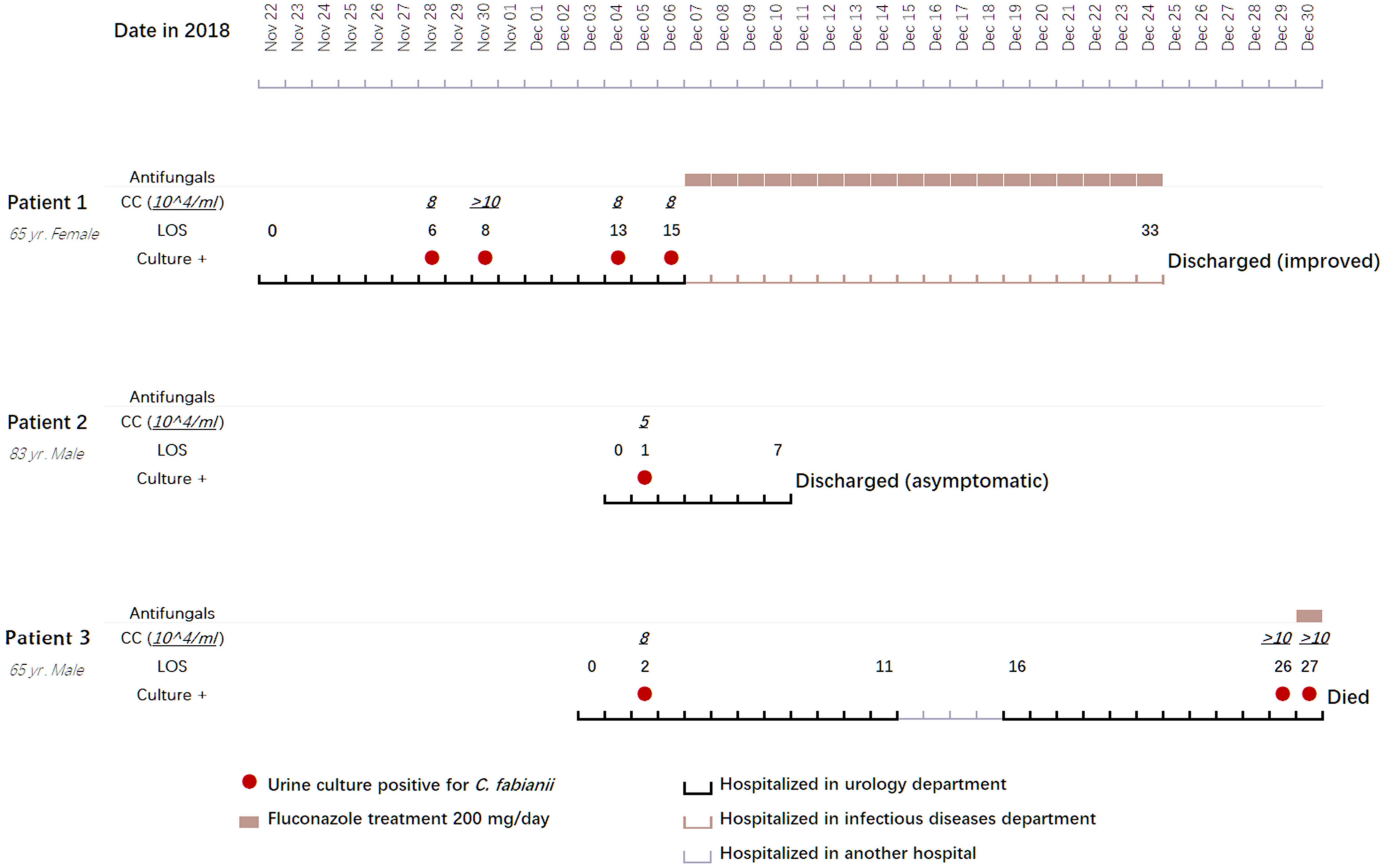
Clinical features, treatment regimens, and outcomes of three clustered cases with *Cyberlindnera fabianii* funguria. Abbreviations: CC, CFU (colony forming unit) count; LOS, length of stay; Culture +: culture positive for *C. fabianii*.

*C. fabianii* strains were isolated from urine samples of three patients (number 1-3); on four different occasions for patient number 1, only once for patient number 2, and on three different occasions for patient number 3 (**Figure 1** and **Table 1**). After these three cases, no further *C. fabianii* cases were detected in the same hospital for over seven months, till a new *C. fabianii* isolate, cultured from an ascites sample of a patient admitted to general surgery department (recorded as patient number 4), was detected, and this isolate was used as a comparator.

**Table 1:** Clinical features of four patients with *Cyberlindnera fabianii* funguria and microbiology characteristics of the strains.

Patient	No. 1	No. 2	No. 3	No. 4
Patient features				
Reason for hospitalization	Fever and parastomal fistula	Postoperative follow-up of bladder cancer	Fever with backaches	Pancreatic cancer
Underlying disease	Bladder cancer	Bladder cancer	Bladder cancer, diabetes	No
Clinical status at time of first positive culture				
Fever	Yes	No	Yes	Yes
Immunosuppressive state	Yes	Yes	Yes	Yes
Neutropenia (<10^9^/L)	No	No	No	No
High urine leukocytes	Yes	No	Yes	No
Prior antibacterial exposure	Yes	Yes	Yes	Yes
Prior antifungal exposure	No	No	No	No
Abdominal surgery	Yes	Yes	Yes	Yes
Indwelling urinary catheter	No	No	Yes	No
Parenteral nutrition	No	No	No	Yes
Yeast culture				
Department of hospitalization	Urology	Urology	Urology	General surgery
Samples positive for yeasts	Urine	Urine	Urine	Ascites fluid
Number of times isolated	4	1	3	1
Mixed bacteria culture positive	*Enterococcus faecium*	No	*Enterococcus faecium*	*Enterococcus faecium*, *Enterobacter cloacae*
Identification				
Lab no. of first strain	CYCFB01-1	CYCFB02-1	CYCFB03-1	CYCFB04-1
ITS sequencing	*C. fabianii*	*C. fabianii*	*C. fabianii*	*C. fabianii*
Identity with type strain	100%	100%	100%	100%
Vitek MS	No identification	No identification	No identification	No identification
Antifungal susceptibility (mg/L)				
Fluconazole	1	1	0.5	1
Voriconazole	0.015	0.015	0.015	0.03
Itraconazole	0.12	0.12	0.06	0.06
Posaconazole	0.12	0.12	0.12	0.12
Caspofungin	0.03	0.06	0.03	0.06
Micafungin	0.03	0.03	0.03	0.03
Anidulafungin	0.015	0.06	0.015	0.015
5- Flucytosine	0.12	0.06	0.12	0.06
Amphotericin B	0.5	0.5	0.25	0.25
Data availability				
ITS	OP904191	OP904192	OP904193	OP904194
NTS-1	OP912967	OP912968	OP9129689	OP91296870
WGS	SAMN32011978	SAMN32011979	SAMN320119810	SAMN32011981

ITS, rDNA internal transcribed spacer region; NTS-1, rDNA non-transcribed spacer region-1; WGS, whole genome sequencing.

Routine culture of specimens was carried out as per standard laboratory protocols. Generally, for urine samples, 1 μl of the sample was inoculated on Blood Agar media and incubated at 35 °C for 24 h. Thereafter, the number of colonies growing on the media plate was counted to ensure that they met the criterion for a urinary tract infection. A brief identification protocol revealed that the cultured isolates were yeast. Thus, Sabouraud glucose agar (SDA) was used to subculture these isolates for further identification testing. Attempts were made to identify the cultured yeasts by using a Vitek MS MALDI-TOF MS system (bioMérieux, Marcy l’Etoile, France, with IVD database version 2.1), following manufacturer’s instructions. For each run, *Escherichia coli* strain ATCC 8739 was used to calibrate and control the method. Unfortunately, this system was unable to identify the yeast strains.

### Molecular identification and phylogenetic analysis by rDNA gene spacer regions

As all the suspected yeast isolates could not be identified using the MALDI-TOF MS systems, sequencing of rDNA internal transcribed spacer (ITS) regions was carried out. Briefly, DNA extraction of the isolates was performed using a QIAamp DNA Mini Kit (Qiagen, Hilden, Germany). The universal primer pair ITS1 and ITS4 was used for amplification and sequencing of the ITS region for each strain (Xiao et al., 2014), and a species identification was carried out by querying against the Westerdijk Fungal Biodiversity Institute’s database using a web-based pairwise alignment tool (https://wi.knaw.nl/page/Pairwise_alignment ).

Further, to investigate the potential relatedness of these cases, the first yeast isolate of each patient case was chosen for further testing, and the rDNA non-transcribed spacer region 1 (NTS-1) was amplified with a forward primer NTS1-F (5’-GGGATAAATCATTTGTATACGAC-3’) and a reverse primer NTS1-R (5’-TTGCGGCCATATCCACAAGAAA-3’) as described previously (Al-Sweih et al., 2019), and sequenced from both directions. A phylogenetic tree of NTS-1 sequences was generated by Mega X (version 10.2, https://www.megasoftware.net/ ) using neighbor-joining method with bootstrap value of 1000. NTS-1 sequences from *C. fabianii* type strain CBS 5640T, and *C. fabianii* reference genome strain JOY008, were also downloaded from GenBank and included in the analysis. In addition, NTS-1 sequence extracted from the genome of *Cyberlindnera jadinii* strain NBRC 0988 was selected as an outgroup.

### Whole genome sequencing and analysis of *C. fabianii* strains

Whole-genome sequencing was performed on each of the first yeast strain from each of patients 1 to 4. Generally, a 350-bp DNA library was prepared using NEB Next Ultra DNA library prep kits (NEW ENGLAND BioLabs Inc., MA, USA), following the manufacturer’s instructions. Library integrity was evaluated with an Agilent 2100 Bioanalyzer (Agilent Technologies, CA, USA). Sequencing was performed on an Illumina NovaSeq using PE150 in a commercial company (Novogene Co., Ltd., Beijing, China).

For genome analysis, the complete reference genome of *C. fabianii* strain JOY008 (GenBank accession no. GCA_022641835.1) was used for read mapping. Single-nucleotide polymorphism (SNP) analysis was carried out by Burrows-Wheeler Aligner (version 0.7.7), SAMtools (version 1.2), and Genome Analysis Toolkit (GATK) (v.3.3-0) per GATK Best Practices (Li and Durbin, 2009; Li et al., 2009; Mckenna et al, 2010). The filtered reads were compared to the reference genome by SAMtools to generate BAM files. Then, variants were marked by GATK MarkDuplicates for each sample, and single-sample GVCF files were created by GATK HaplotypeCaller with the option –emitRefConfidence GVCF. The GVCF files were aggregated by GATK CombineGVCFs tool. After that, the GVCF files were jointly genotyped with the GATK GenotypeGVCFs to produce a single VCF file containing variants data on every strain. Finally, the VCF file was selected using GATK SelectVariants with the option -select-type SNP and filtered using the following parameters: VariantFiltration, QD < 2.0, ReadPosRankSum < −8.0, FS > 60.0, MQRankSum < −12.5, MQ < 40.0 and HaplotypeScore > 13.0.

Specifically, in this study, the term “pseudo-outbreak” was used to describe inappropriate artifactual clustering of real infections as an outbreak event, due to limitation of investigation tools.

### Antifungal susceptibility testing

Minimum inhibitory concentrations (MICs) of all the isolates were determined by Sensititre YeastOne YO10 kits (Thermo Scientific, OH, USA) following the manufacturer’s instructions, and with *Candida krusei* ATCC 6258 and *Candida parapsilosis* ATCC 22019, used as quality control strains.

### Data availability

DNA sequences of rDNA ITS and NTS-1 regions for each of the first yeast strain isolated from each individual has been deposited into NCBI GenBank database (accession nos. OP904191-OP904194 for ITS region and OP912967-OP912970 for NTS-1 region). Their WGS reads data is also now available in National Microbiology Data Center (NMDC) database (Bioproject accession no. PRJNA907923).

## Results

### Patients

Information pertaining to each of the 4 patients included in this case study is summarized in **Figure 1** and **Table 1**.

Patient 1 was a 65-year-old female admitted to the urology department of Beijing Chao-Yang hospital on Nov 22, 2018, due to presence of fever for two weeks and a parastomal fistula after ileal replacement due to bladder cancer in 2016. Upon admission, the patient had fever for over a week. On day 6 after admission, a yeast strain was isolated from her urine sample and the colony count (CC) was 8×10^4^ CFU/ml. The same urine culture also grew *Enterococcus faecium* (5×10^4^ CFU/ml) as a mixed culture with the yeast. The routine MALDI-TOF MS identification system failed to identify the yeast isolate. Her urine samples collected on days 8, 13 and 15 after admission also yielded yeasts (CC of 8 to >10×10^4^ CFU/ml). Follow-up ITS sequencing assigned all the strains as *C. fabianii*. The patient was given fluconazole at 200 mg/day for 18 days after which her condition improved notably, and she was finally discharged from the hospital on day 33 of admission.

Patient 2 was an 83-year-old male admitted to the urology department of the hospital on Dec 04, 2018, for follow-up of bladder cancer electrosurgery performed eight and four months before his admission. On day 1 after admission, a urine sample was collected for routine screening, which was reported positive for yeasts with a CC of 5×10^4^ CFU/ml. The yeast strain was identified as *C. fabianii* by ITS sequencing. This patient didn’t present with any symptoms of infection, and hence antifungal therapy was not given. Later, he received a transurethral resection of bladder tumor on day 3, and was discharged on day 7 after admission.

Patient 3 was a 65-year-old male admitted to the urology department of the hospital on Dec 03, 2018, due to presence of high fever with backaches. Nine months before this admission, the patient hand undergone nephroureterectomy of the left kidney. He received nephrostomy on the right kidney immediately on day 1 after admission. His urine sample collected on day 2 was culture positive for a yeast (CC: 8×10^4^ CFU/ml), which was identified as *C. fabianii* by ITS sequencing. However, no antifungal agents were prescribed for him and only a broad-spectrum antibiotic was given. On days 26 and 27, two urine samples were collected consecutively and both were positive for *C. fabianii* with a CC of > 10×10^4^ CFU/ml. Of note, all his urine samples also grew *E. faecium* (>10×10^4^ CFU/ml) as part of a mixed culture with the yeast. Though fluconazole therapy (200 mg/day) was initiated on day 27 after admission, the patient passed away on the same day.

Patient 4 was a 54-year-old female admitted to the general surgery department of the hospital on Jul 18, 2019, which was over seven months after the patient 1, 2 and 3 case clusters. She was hospitalized due to pancreatic cancer, and received radical pancreatoduodenectomy on day 12 after admission; later with pancreatic intestinal anastomotic fistula. The patient’s ascites sample collected on day 20 was reported positive for *C. fabianii* and *E. faecium.* However, she didn’t have any other culture positive results for fungi after that, nor received any antifungal treatment, and was discharged from the hospital on day 52.

### *C. fabianii* identification

All the yeast strains could not be identified using the Vitek MS MALDI-TOF MS system IVD 2.0 database, nor were the isolates misidentified as something else (identification confidence values <60.0). This is not surprising as *C. fabianii* is not currently included in the system’s spectrum database.

By sequencing of the ITS region, all the yeast strains from the four patients were unambiguously assigned to *C. fabianii*, with their respective ITS sequences 100% (602/602 bp) identical to that of *C. fabianii* type strain CBS 5640T.

### Phylogenetic analysis by rDNA NTS-1 region

Since *C. fabianii* is a rare yeast species identified in human patients, and the fact that the clustered cases (patients 1 to 3) described here were identified within a two-week period from the same department, an investigation was carried out to assess the possibility of this being a nosocomial outbreak. Owing to the high sequence similarity of the ITS region among the strains, sequence analysis based on rDNA NTS-1 region was further carried out, which was assumed to have higher discriminatory power and has previously been used to confirm a *C. fabianii* outbreak in Kuwait (Al-Sweih et al., 2019).

**Figure 2:**
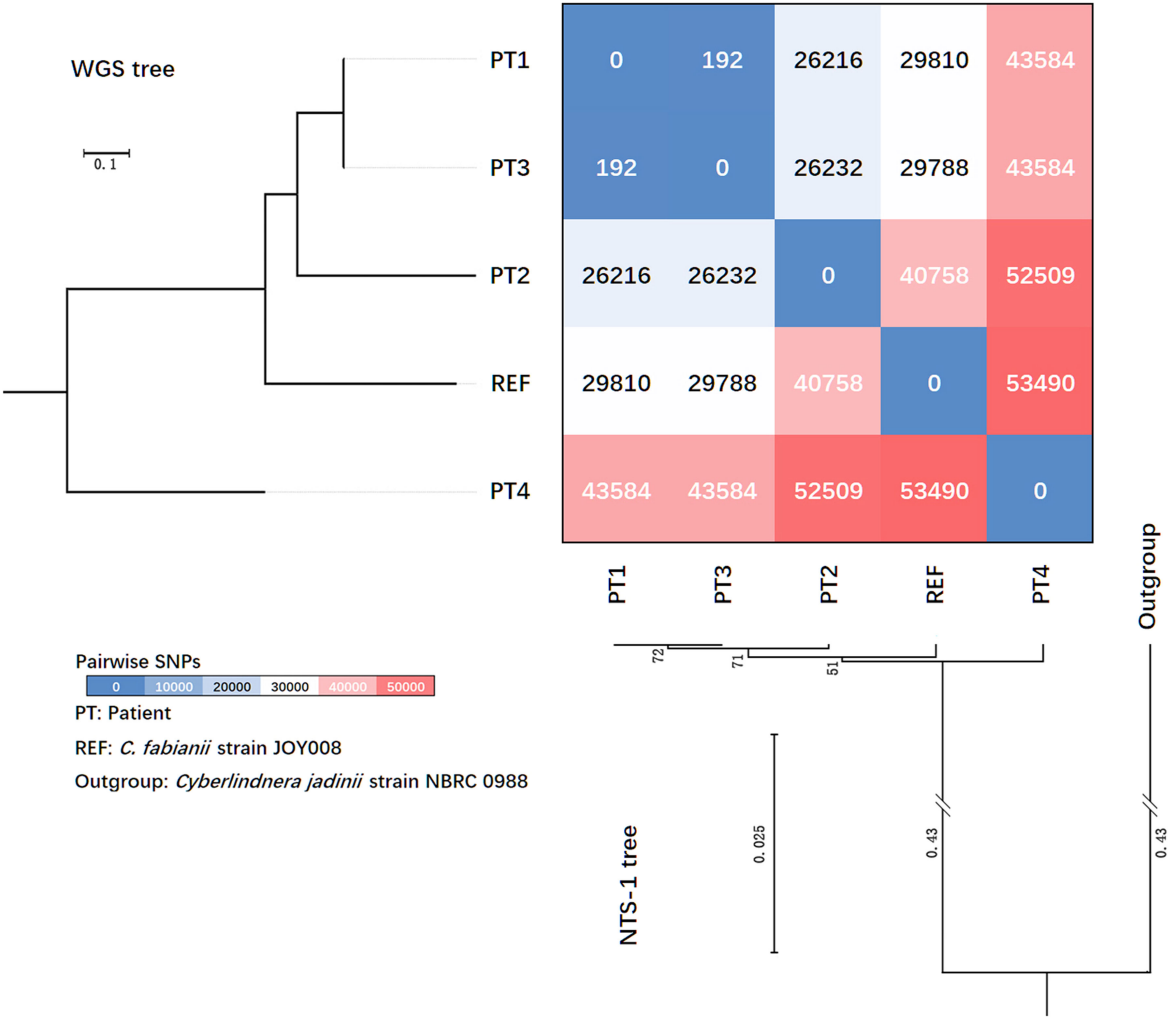
Phylogenetic trees generated based on rDNA non-transcribed spacer (NTS) region-1 sequences and whole genome sequencing (WGS) SNPs, and heatmaps revealing pairwise differences of SNPs amongst four patients’ strains collected in this study.

Using *C. jadinii* (strain NBRC 0988) as an outgroup, the phylogenetic tree based on the NTS-1 region clustered all the *C. fabianii* isolates together (**Figure 2**), and inter-species variation between *C. jadinii* and *C. fabianii* in NTS-1 region was >43.5%. Amongst *C. fabianii* strains, some intra-species variation was observed (**Figure 2**). However, sequence variations amongst the strains from patients 1 to 3 was inconspicuous, as these strains exhibited the same sequence type, while the strain from patient 2 had only one SNP (identity 1179/1180, 99.92%). In contrast, strains from the three clustered cases were quite diverged from the strain from patient 4 which was isolated seven months later, which had an overall 7-bp insertions and 4 additional SNPs in its NTS-1 region (identity 99.07%).

### Whole genome sequencing results

Genome sequencing of yeast strains from patients 1 to 4 produced 2.6 to 3.7 Gb of clean data, and average depths of sequencing were all above 200×. The average genome size obtained was 12.94 Mb. Their genomes had an average GC content of 44.4% to 45.1%, with N50 of 13,739 bp to 202,514 bp. Comparative genomic analysis was performed for all strains. The pairwise differences between genome reference strain JOY008, which originated from a soil environment in USA, and our four patients’ clinical strains, were 29,810-53,490 SNPs (**Figure 2**).

We carried out a review of previous outbreak reports caused by different yeast species, and the number of pairwise SNPs described varied from less than ten to several hundred (**Table 2**). The yeast strains from patient 1 and 3 had only 192 SNPs identified, suggesting that they were probably closely related (**Figure 2**). However, there were over 26,000 SNPs identified between the strain from patient 2 and strains from patients 1 and 3 (**Figure 2**), which was considered as a high-level genomic variation. These findings suggested that the *C. fabianii* strain from patient 2 was from a different origin. In addition, the yeast strain from patient 4 was even more diverged, with >43,000 of SNPs compared to all strains from patients 1 to 3, and the reference genome (**Figure 2**). Lastly, the phylogenetic tree constructed based on whole genome SNPs also support the same conclusion (**Figure 2**).

**Table 2:** Review of outbreak events caused by yeast species that were characterized by whole genome sequencing in previous studies.

Species	Reference Genome size (Mb)	Country	Patient population	Ward	No. of cases with WGS	No. of SNPs within each event	Reference
*Candida albicans*	14.3	Spain	Neonate	ICU	2-11	134-769	(Guinea et al., 2021)
*Candida parapsilosis*	13	Spain	Neonate	ICU	2-4	49-241	(Guinea et al., 2021)
*Candida auris*	12.7	US	Adults	Not specified	26	2-50	(De St Maurice et al., 2022)
		India	Adults	Medical wards	2-2	≤7	(Yadav et al., 2021)
		UK	Adults	ICU, high dependency units, surgical admission ward	5-17	≤134	(Rhodes et al., 2018)
		UK	Adults	ICU, neurosciences wards	37	≤215	(Eyre et al., 2018)
		Colombia	Not specified	Not specified	5	≤40	(Escandon et al., 2019)
		USA	Not specified	Not specified	10	≤12	(Chow et al., 2018)
*Dirkmeia churashimaensis*	21	India	Neonate	ICU	6	1,621	(Chowdhary et al., 2020a)
*Candida blankii*	14.8	India	Neonate	ICU	6	≤277	(Chowdhary et al., 2020b)
*Malassezia pachydermatis*	8.2	USA	Neonate	ICU	5	≤14	(Chow et al., 2020)
*Cyberlindnera fabianii*	12.3	China	Adults	Urology department	2	192	This study

### Antifungal susceptibilities

All the *C. fabianii* strains isolated in this study showed good susceptibility to all the nine antifungal agents tested (**Table 1**), with geometric minimum inhibitory concentration (GM MIC) of 0.84 mg/L to fluconazole, 0.02 mg/L to voriconazole, 0.08 mg/L to itraconazole, 0.12 mg/L to posaconazole, 0.04 mg/L to caspofungin, 0.03 mg/L to micafungin, 0.02 mg/L to anidulafungin, 0.08 mg/L to 5-flucytosine, and finally, 0.35 mg/L to amphotericin B. If using clinical breakpoints or epidemiological cut-off values of *C. albicans* as references, all these strains could be classified as susceptible or of wild-type phenotype to all antifungal agents tested.

## Discussion

*C. fabianii*, basionym *Hansenula fabianii*, homotypic synonyms *Candida fabianii*, *Lindnera fabianii* and *Pichia fabianii*, is an ascomycetous yeast that has a close relationship with human activities (Kato et al., 1997; Arastehfar et al., 2019; Van Rijswijck et al., 2019). This yeast species is commonly seen in fermented food products like alcohols (Arastehfar et al., 2019; Van Rijswijck et al, 2019), and has also been used for treatment of waste water with a long history (Kato et al., 1997). The species has now been assigned within the Wickerhamomycetaceae clade (Kidd et al., 2023). Within this clade, there are several other species that have been reported to cause human infections, such as *Wickerhamomyces anomalus* and *Cyberlindnera jadinii* (Treguier et al., 2018; Zhang et al., 2021).

Generally, detection of *C. fabianii* in clinical settings is rare. According to previous surveillance reports on human fungal diseases, the prevalence of *C. fabianii* is generally <0.1% (Pfaller et al., 2019; Xiao et al., 2020). However, this yeast species is also an opportunistic pathogen that can cause a broad-range of infections, including lethal fungemia (Al-Sweih et al., 2019; Arastehfar et al., 2019). A previous research suggests that *C. fabianii* only has low virulence attributes (Arastehfar et al., 2019), although Nouraei et al. observed that this fungal species was one of the uncommon yeasts with high-level production of hemolysin, phospholipase and proteinase (Nouraei et al., 2020). In addition, *C. fabianii* has been observed to have a strong capacity for biofilm formation, which may contribute to its persistence and resistance to antifungal therapies (Hamal et al., 2008; Nouraei et al., 2020).

Of note, several studies have revealed difficulties in the accurate identification of *C. fabianii* using conventional methods, which may influence precision clinical recognition and management of infections caused by this organism (Svobodova et al., 2016; Al-Sweih et al., 2019). MALDI-TOF MS has been reported as a powerful tool for identification of yeasts, but the system’s identification capacity largely relies on the peptide mass fingerprint database that is incorporated into the system. Some of the MALDI-TOF MS systems, such as Biotyper (Bruker Daltonics, Germany, with IVD library version 8) and MicroIDSys (ASTA, Korea, with database version 1.23.2), have demonstrated capacity to accurately identify *C. fabianii* strains (Park et al., 2019; Teke et al., 2021). In contrast to this, *C. fabianii* is still absent from the Vitek MS IVD database (up to version 3.2), hence this system failed to identify any of *C. fabianii* isolates in this study. Similar findings were also reported by Teke et al. (Teke et al., 2021).

Although nosocomial transmission of fungal pathogens is less frequently encountered in clinical practice compared to bacterial pathogens, fungal outbreaks are more common than publicly appreciated, and are mostly associated with medical products or contamination of the hospital environment (Kanamori et al., 2015; Litvintseva et al., 2015; Magill et al., 2018). For instance, *Candida parapsilosis*, one of the most prevalent human pathogenic yeast species, is well-known for causing catheter-related bloodstream infections. There have been a large number of nosocomial outbreaks caused by *Candida parapsilosis* worldwide, including several recently reported cases caused by fluconazole-resistant clones that raised more public health concerns (Arastehfar et al., 2020; Zhang et al., 2020; Thomaz et al., 2022). Moreover, reports of outbreaks caused by unusual fungal pathogens, such as the recently emerged *C. auris*, are increasing (Litvintseva et al., 2015; Chow et al., 2018). Of note, during the COVID-19 pandemic period, fungal outbreaks caused higher medical burdens to healthcare facilities and patients (Hoenigl et al., 2022; Thomaz et al., 2022), and hence are beginning to receive more attention.

Of note, a recent outbreak of *C. fabianii* in Kuwait was described by Al-Sweih et al., which involved a total of 10 fungemia cases in neonates (Al-Sweih et al., 2019). Furthermore, previous reviews on *C. fabianii* cases have demonstrated that the elderly population is the second most vulnerable population after neonates overall, with funguria being the first to second commonest clinical symptom (Al-Sweih et al., 2019; Arastehfar et al., 2019; Park et al., 2019). This agrees with our three-clustered *C. fabianii* funguria cases which all occurred in elderly patients, and with funguria as the common clinical symptom, though not every patient had symptomatic urinary tract infection.

Published literature have emphasized that presence of indwelling urinary catheter is the most important risk factor and transmission route for nosocomial urinary tract infections, especially when catheter care quality is poor. However, a variety of additional risk factors have also been described, including female gender, increased age, diabetes, bladder instrumentation, urinary outflow obstruction, amongst others. (Pearson-Stuttard et al., 2016; Mody et al., 2017; Odabasi and Mert, 2020). Of the four patients described in this study, only one carried an indwelling urinary catheter, and all of them had undergone abdominal surgeries prior to the onset of funguria. Besides, three of the four patients had *E. faecium* detected concurrently with *C. fabianii* in the same urine sample. *Enterococcus* species, including *E. faecium*, are well-known ubiquitous inhabitants of the human gut microbiota and could lead to urinary tract infections (Magruder et al., 2019). Moreover, *C. fabianii* has also been identified in the human intestinal microbiota (Zhai et al., 2020), and previously Mathy et al. hypothesized that translocation of *C. fabianii* from the gut was responsible for a ventriculoperitoneal shunt case (Mathy et al., 2020). Therefore, it is possible that *C. fabianii* funguria cases identified in our study may have resulted from gut microbiota translocations, and abdominal surgeries might serve as triggers or risk factors.

As widely-acknowledged, application of ITS sequencing could allow accurate identification of yeast species but with insufficient discriminatory power for intra-species typing (Stielow et al., 2015; Al-Sweih et al., 2019). Al-Sweih et al. applied sequencing of NTS-1 regions, a gene locus that is considered to have a higher discriminatory power, in *C. fabianii* outbreak investigation, and found that all outbreak strains in Kuwait shared 100% identical NTS-1 sequences (Al-Sweih et al., 2019). In comparison, we found a single SNP within NTS-1 region on patient 2’s strain versus strains from patients 1 and 3 in this study. However, further solid evidence was still needed to rule out the possibility of a potential outbreak.

To address concerns on readiness and limitations in discriminatory power of molecular typing methods in outbreak investigations, WGS has been recommended as a valuable alternative (Litvintseva et al., 2015; Bougnoux et al., 2018; Desnos-Ollivier et al., 2020). In this study, SNP-based analysis based on results acquired from WGS data clearly suggested that the genome of patient 2’s strain was quite divergent amongst the three clustered cases, which indicated a pseudo-outbreak event. Of note, the phrase “pseudo-outbreaks” could refer to either clustering of false infections, or artifactual clustering of real infections (Wallace et al., 1998). Clustering of false infections was more widely-noted, which may be associated with e.g. medical device or clinical laboratory contaminations (Kirby et al., 2017; Abdolrasouli et al., 2021). However, as indicated in our study, artifactual misinterpretation of “outbreaks” due to limitation of investigation methodologies (such as inadequate discriminatory power of molecular typing assays) should also be avoided.

Although WGS has made significant contributions in epidemiological studies, some limitations still remain. One major issue, as noted in outbreak investigations of all microbes including bacteria and fungi, is lack of consensus for data interpretation. Specifically, setting-up pairwise SNP-based cut-off values for assigning transmission events is still cumbersome, which has limited the wide utility of WGS in epidemiological studies (Coll et al., 2020; Guinea et al., 2021). In review of previous reports for outbreaks caused by yeast species that were characterized by WGS, it can be seen that the number of pairwise SNPs described in different studies of diverged species varied significantly, from less than ten to over hundreds. In the present study, genomic evidence clearly supported that patient 2’s *C. fabianii* strain was from a different origin, compared to others (with >26,000 SNPs compared to strains from patients 1 and 3). However, the 192 pairwise-SNP between strains from patient 1 and 3 may suggest that these two patients could have acquired the yeasts from a common source in the same ward but through different routes, rather than a direct transmission between the 2 patients, in which case the number of SNPs would be expected to be much less. But the hypothesis needs additional evaluation in a larger population and with more cases.

Due to the possibility of nosocomial transmission of this yeast in the described ward, surveillance infection control cultures were obtained to screen for *C. fabianii* in the department’s environment and amongst related health-care staff, but no *C. fabianii* was detected. Additional infection control strategies implemented further included enhancing environmental cleaning and hand hygiene practices, as well as providing education of fungal nosocomial infections to all healthcare staff.

One limitation of the study is that, antifungal susceptibility testing was not carried out using the standard broth microdilution methods, though YeastOne has proved equally efficient with good correlation in testing of yeasts (Cuenca-Estrella et al., 2010). Furthermore, with the limited number of cases studied, our base-line understanding for intra-species variation of *C. fabianii* genomes was still limited. Interpretation of any outbreak events shouldn’t simply rely on WGS result alone. It warrants a comprehensive analysis of different aspects of the cases, including patients’ clinical characteristics and epidemiological data, as well as the pathogens’ phenotypic and molecular characteristics.

In conclusion, as there are increasing reports of nosocomial outbreaks caused by emerging and uncommon fungal species, increased awareness of these rare organisms is warranted in public health. Conventional genotyping methods may have limited discriminatory power in investigating outbreaks due to these rare organisms; WGS has proven to be a good typing method for supporting investigation of such rare outbreak events.

## Data availability statement

The datasets presented in this study can be found in online repositories. The names of the repository/repositories and accession number(s) can be found below: Genbank: OP904191-OP904194 for ITS region, OP912967-OP912970 for NTS-1 region. WGS reads data can be found in NCBI database under Bioproject accession no. PRJNA907923.

## Ethics statement

The studies involving human participants were reviewed and approved by Human Research Ethics Committee of the Beijing Chaoyang Hospital. Written informed consent for participation was not required for this study in accordance with the national legislation and the institutional requirements.

## Author contributions

XF, R-CD and LG conceived the work. XF, and R-CD performed the experiments and data analysis. XF, TK, and LG drafting the manuscript. All authors contributed to the article and approved the submitted version.

## References

[B1] Abdolrasouli A. Gibani M. M. De Groot T. Borman A. M. Hoffman P. Azadian B. S.. (2021). A pseudo-outbreak of rhinocladiella similis in a bronchoscopy unit of a tertiary care teaching hospital in London, united kingdom. Mycoses 64, 394–404. doi: 10.1111/myc.13227 33314345

[B2] Al-Sweih N. Ahmad S. Khan S. Joseph L. Asadzadeh M. Khan Z. (2019). Cyberlindnera fabianii fungaemia outbreak in preterm neonates in Kuwait and literature review. Mycoses 62, 51–61. doi: 10.1111/myc.12846 30184277

[B3] Arastehfar A. Daneshnia F. Hilmioglu-Polat S. Fang W. Yasar M. Polat F.. (2020). First report of candidemia clonal outbreak caused by emerging fluconazole-resistant candida parapsilosis isolates harboring Y132F and/or Y132F+K143R in Turkey. Antimicrob. Agents Chemother. 64, e01001–20. doi: 10.1128/AAC.01001-20 32690638 PMC7508617

[B4] Arastehfar A. Fang W. Al-Hatmi A. M. S. Afsarian M. H. Daneshnia F. Bakhtiari M.. (2019). Unequivocal identification of an underestimated opportunistic yeast species, cyberlindnera fabianii, and its close relatives using a dual-function PCR and literature review of published cases. Med. Mycol 57, 833–840. doi: 10.1093/mmy/myy148 30649481 PMC6739237

[B5] Bougnoux M. E. Brun S. Zahar J. R. (2018). Healthcare-associated fungal outbreaks: New and uncommon species, new molecular tools for investigation and prevention. Antimicrob. Resist. Infect. Control 7, 45. doi: 10.1186/s13756-018-0338-9 29599969 PMC5870726

[B6] Chen S. C. Perfect J. Colombo A. L. Cornely O. A. Groll A. H. Seidel D.. (2021). Global guideline for the diagnosis and management of rare yeast infections: an initiative of the ECMM in cooperation with ISHAM and ASM. Lancet Infect. Dis. 21, e375–e386. doi: 10.1016/S1473-3099(21)00203-6 34419208

[B7] Chow N. A. Chinn R. Pong A. Schultz K. Kim J. Gade L.. (2020). Use of whole-genome sequencing to detect an outbreak of malassezia pachydermatis infection and colonization in a neonatal intensive care unit-california-2016. Infect. Control Hosp Epidemiol. 41, 851–853. doi: 10.1017/ice.2020.73 32370815

[B8] Chow N. A. Gade L. Tsay S. V. Forsberg K. Greenko J. A. Southwick K. L.. (2018). Multiple introductions and subsequent transmission of multidrug-resistant candida auris in the USA: a molecular epidemiological survey. Lancet Infect. Dis. 18, 1377–1384. doi: 10.1016/S1473-3099(18)30597-8 30293877 PMC6556114

[B9] Chowdhary A. Sharada K. Singh P. K. Bhagwani D. K. Kumar N. De Groot T.. (2020a). Outbreak of dirkmeia churashimaensis fungemia in a neonatal intensive care unit, India. Emerg. Infect. Dis. 26, 764–768. doi: 10.3201/eid2604.190847 32186501 PMC7101094

[B10] Chowdhary A. Stielow J. B. Upadhyaya G. Singh P. K. Singh A. Meis J. F. (2020b). Candida blankii: an emerging yeast in an outbreak of fungaemia in neonates in Delhi, India. Clin. Microbiol. Infect. 26 648, e645–648e648. doi: 10.1016/j.cmi.2020.01.001 31927114

[B11] Coll F. Raven K. E. Knight G. M. Blane B. Harrison E. M. Leek D.. (2020). Definition of a genetic relatedness cutoff to exclude recent transmission of meticillin-resistant staphylococcus aureus: a genomic epidemiology analysis. Lancet Microbe 1, e328–e335. doi: 10.1016/S2666-5247(20)30149-X 33313577 PMC7721685

[B12] Cuenca-Estrella M. Gomez-Lopez A. Alastruey-Izquierdo A. Bernal-Martinez L. Cuesta I. Buitrago M. J.. (2010). Comparison of the vitek 2 antifungal susceptibility system with the clinical and laboratory standards institute (CLSI) and European committee on antimicrobial susceptibility testing (EUCAST) broth microdilution reference methods and with the sensititre YeastOne and etest techniques for *in vitro* detection of antifungal resistance in yeast isolates. J. Clin. Microbiol. 48, 1782–1786. doi: 10.1128/JCM.02316-09 20220169 PMC2863906

[B13] Desnos-Ollivier M. Maufrais C. Pihet M. Aznar C. Dromer F. French Mycoses Study G. (2020). Epidemiological investigation for grouped cases of trichosporon asahii using whole genome and IGS1 sequencing. Mycoses 63, 942–951. doi: 10.1111/myc.13126 32506754

[B14] De St Maurice A. Parti U. Anikst V. E. Harper T. Mirasol R. Dayo A. J.. (2022). Clinical, microbiological, and genomic characteristics of clade-III candida auris colonization and infection in southern california-2022. Infect. Control Hosp Epidemiol. 1-9. doi: 10.1017/ice.2022.204 PMC10369217 36052507

[B15] Escandon P. Chow N. A. Caceres D. H. Gade L. Berkow E. L. Armstrong P.. (2019). Molecular epidemiology of candida auris in Colombia reveals a highly related, countrywide colonization with regional patterns in amphotericin B resistance. Clin. Infect. Dis. 68, 15–21. doi: 10.1093/cid/ciy411 29788045

[B16] Eyre D. W. Sheppard A. E. Madder H. Moir I. Moroney R. Quan T. P.. (2018). And its control in an intensive care setting. N Engl. J. Med. 379, 1322–1331. doi: 10.1056/NEJMoa1714373 30281988

[B17] Guinea J. Mezquita S. Gomez A. Padilla B. Zamora E. Sanchez-Luna M.. (2021). Whole genome sequencing confirms candida albicans and candida parapsilosis microsatellite sporadic and persistent clones causing outbreaks of candidemia in neonates. Med. Mycol 60. doi: 10.1093/mmy/myab068 34718724

[B18] Hamal P. Ostransky J. Dendis M. Horvath R. Ruzicka F. Buchta V.. (2008). A case of endocarditis caused by the yeast pichia fabianii with biofilm production and developed *in vitro* resistance to azoles in the course of antifungal treatment. Med. Mycol 46, 601–605. doi: 10.1080/13693780802078180 18608935

[B19] Hoenigl M. Seidel D. Sprute R. Cunha C. Oliverio M. Goldman G. H.. (2022). COVID-19-associated fungal infections. Nat. Microbiol. 7, 1127–1140. doi: 10.1038/s41564-022-01172-2 35918423 PMC9362108

[B20] Kanamori H. Rutala W. A. Sickbert-Bennett E. E. Weber D. J. (2015). Review of fungal outbreaks and infection prevention in healthcare settings during construction and renovation. Clin. Infect. Dis. 61, 433–444. doi: 10.1093/cid/civ297 25870328

[B21] Kato M. Iefuji H. Miyake K. Iimura Y. (1997). Transformation system for a wastewater treatment yeast, hansenula fabianii J640: isolation of the orotidine-5’-phosphate decarboxylase gene (URA3) and uracil auxotrophic mutants. Appl. Microbiol. Biotechnol. 48, 621–625. doi: 10.1007/s002530051105 9421925

[B22] Kidd S. E. Abdolrasouli A. Hagen F. (2023). Fungal nomenclature: Managing change is the name of the game. Open Forum Infect. Dis. 10, ofac559. doi: 10.1093/ofid/ofac559 36632423 PMC9825814

[B23] Kirby J. E. Branch-Elliman W. Lasalvia M. T. Longhi L. Mackechnie M. Urman G.. (2017). Investigation of a candida guilliermondii pseudo-outbreak reveals a novel source of laboratory contamination. J. Clin. Microbiol. 55, 1080–1089. doi: 10.1128/JCM.02336-16 28100597 PMC5377835

[B24] Li H. Durbin R. (2009). Fast and accurate short read alignment with burrows-wheeler transform. Bioinformatics 25, 1754–1760. doi: 10.1093/bioinformatics/btp324 19451168 PMC2705234

[B25] Li H. Handsaker B. Wysoker A. Fennell T. Ruan J. Homer N.. (2009). The sequence Alignment/Map format and SAMtools. Bioinformatics 25, 2078–2079. doi: 10.1093/bioinformatics/btp352 19505943 PMC2723002

[B26] Litvintseva A. P. Brandt M. E. Mody R. K. Lockhart S. R. (2015). Investigating fungal outbreaks in the 21st century. PloS Pathog. 11, e1004804. doi: 10.1371/journal.ppat.1004804 25996829 PMC4440772

[B27] Magill S. S. O’leary E. Janelle S. J. Thompson D. L. Dumyati G. Nadle J.. (2018). Changes in prevalence of health care-associated infections in U.S. hospitals. N Engl. J. Med. 379, 1732–1744. doi: 10.1056/NEJMoa1801550 30380384 PMC7978499

[B28] Magruder M. Sholi A. N. Gong C. Zhang L. S. Edusei E. Huang J.. (2019). Gut uropathogen abundance is a risk factor for development of bacteriuria and urinary tract infection. Nat. Commun. 10, 5521. doi: 10.1038/s41467-019-13467-w 31797927 PMC6893017

[B29] Mathy V. Chousterman B. Munier A. L. Cambau E. Jacquier H. De Ponfilly G. P. (2020). First reported case of postneurosurgical ventriculoperitonitis due to kocuria rhizophila following a ventriculoperitoneal shunt placement. Infect. Dis. Clin. Pract. 28, 169–170. doi: 10.1097/IPC.0000000000000829

[B30] Mckenna A. Hanna M. Banks E. Sivachenko A. Cibulskis K. Kernytsky A.. (2010). The genome analysis toolkit: a MapReduce framework for analyzing next-generation DNA sequencing data. Genome Res. 20, 1297–1303. doi: 10.1101/gr.107524.110 20644199 PMC2928508

[B31] Mikosz C. A. Smith R. M. Kim M. Tyson C. Lee E. H. Adams E.. (2014). Fungal endophthalmitis associated with compounded products. Emerg. Infect. Dis. 20, 248–256. doi: 10.3201/eid2002.131257 24447640 PMC3901475

[B32] Mody L. Greene M. T. Meddings J. Krein S. L. Mcnamara S. E. Trautner B. W.. (2017). A national implementation project to prevent catheter-associated urinary tract infection in nursing home residents. JAMA Intern. Med. 177, 1154–1162. doi: 10.1001/jamainternmed.2017.1689 28525923 PMC5710434

[B33] Nouraei H. Pakshir K. Zareshahrabadi Z. Zomorodian K. (2020). High detection of virulence factors by candida species isolated from bloodstream of patients with candidemia. Microb. Pathog. 149, 104574. doi: 10.1016/j.micpath.2020.104574 33075515

[B34] Odabasi Z. Mert A. (2020). Candida urinary tract infections in adults. World J. Urol 38, 2699–2707. doi: 10.1007/s00345-019-02991-5 31654220

[B35] Oliveira M. Azevedo L. (2022). Molecular markers: An overview of data published for fungi over the last ten years. J. Fungi (Basel) 8, 803. doi: 10.3390/jof8080803 36012792 PMC9410331

[B36] Pappas P. G. Lionakis M. S. Arendrup M. C. Ostrosky-Zeichner L. Kullberg B. J. (2018). Invasive candidiasis. Nat. Rev. Dis. Primers 4, 18026. doi: 10.1038/nrdp.2018.26 29749387

[B37] Park J. H. Oh J. Sang H. Shrestha B. Lee H. Koo J.. (2019). Identification and antifungal susceptibility profiles of cyberlindnera fabianii in Korea. Mycobiology 47, 449–456. doi: 10.1080/12298093.2019.1651592 32010466 PMC6968713

[B38] Pearson-Stuttard J. Blundell S. Harris T. Cook D. G. Critchley J. (2016). Diabetes and infection: assessing the association with glycaemic control in population-based studies. Lancet Diabetes Endocrinol. 4, 148–158. doi: 10.1016/S2213-8587(15)00379-4 26656292

[B39] Pfaller M. A. Diekema D. J. Turnidge J. D. Castanheira M. Jones R. N. (2019). Twenty years of the SENTRY antifungal surveillance program: Results for *Candida* species from 1997-2016. Open Forum Infect. Dis. 6, S79–S94. doi: 10.1093/ofid/ofy358 30895218 PMC6419901

[B40] Pulcrano G. Roscetto E. Iula V. D. Panellis D. Rossano F. Catania M. R. (2012). MALDI-TOF mass spectrometry and microsatellite markers to evaluate candida parapsilosis transmission in neonatal intensive care units. Eur. J. Clin. Microbiol. Infect. Dis. 31, 2919–2928. doi: 10.1007/s10096-012-1642-6 22644055

[B41] Reiss E. Tanaka K. Bruker G. Chazalet V. Coleman D. Debeaupuis J. P.. (1998). Molecular diagnosis and epidemiology of fungal infections. Med. Mycol 36 Suppl 1, 249–257. 9988514

[B42] Rhodes J. Abdolrasouli A. Farrer R. A. Cuomo C. A. Aanensen D. M. Armstrong-James D.. (2018). Genomic epidemiology of the UK outbreak of the emerging human fungal pathogen candida auris. Emerg. Microbes Infect. 7, 43. doi: 10.1038/s41426-018-0098-x 29593275 PMC5874254

[B43] Stielow J. B. Levesque C. A. Seifert K. A. Meyer W. Iriny L. Smits D.. (2015). One fungus, which genes? development and assessment of universal primers for potential secondary fungal DNA barcodes. Persoonia 35, 242–263. doi: 10.3767/003158515X689135 26823635 PMC4713107

[B44] Svobodova L. Bednarova D. Ruzicka F. Chrenkova V. Dobias R. Mallatova N.. (2016). High frequency of candida fabianii among clinical isolates biochemically identified as candida pelliculosa and candida utilis. Mycoses 59, 241–246. doi: 10.1111/myc.12454 26763103

[B45] Teke L. Baris A. Bayraktar B. (2021). Comparative evaluation of the bruker biotyper and vitek MS matrix-assisted laser desorption ionization-time of flight mass spectrometry (MALDI-TOF MS) systems for non-albicans candida and uncommon yeast isolates. J. Microbiol. Methods 185, 106232. doi: 10.1016/j.mimet.2021.106232 33961963

[B46] Thomaz D. Y. Del Negro G. M. B. Ribeiro L. B. Da Silva M. Carvalho G. Camargo C. H.. (2022). A Brazilian inter-hospital candidemia outbreak caused by fluconazole-resistant candida parapsilosis in the COVID-19 era. J. Fungi (Basel) 8, 100. doi: 10.3390/jof8020100 35205855 PMC8874954

[B47] Treguier P. David M. Gargala G. Camus V. Stamatoullas A. Menard A. L.. (2018). Cyberlindnera jadinii (teleomorph candida utilis) candidaemia in a patient with aplastic anaemia: a case report. JMM Case Rep. 5, e005160. doi: 10.1099/jmmcr.0.005160 30323936 PMC6152400

[B48] Van Rijswijck I. M. H. Van Mastrigt O. Pijffers G. Wolkers-Rooijackers J. C. M. Abee T. Zwietering M. H.. (2019). Dynamic modelling of brewers’ yeast and cyberlindnera fabianii co-culture behaviour for steering fermentation performance. Food Microbiol. 83, 113–121. doi: 10.1016/j.fm.2019.04.010 31202402

[B49] Wallace R. J. Jr. Brown B. A. Griffith D. E. (1998). Nosocomial outbreaks/pseudo-outbreaks caused by nontuberculous mycobacteria. Annu. Rev. Microbiol. 52, 453–490. doi: 10.1146/annurev.micro.52.1.453 9891805

[B50] World Health Organization (2022). WHO fungal priority pathogens list to guide research, development and public health action. WHO. Available at: https://www.who.int/publications/i/item/9789240060241.

[B51] Xiao M. Chen S. C. Kong F. Xu X. L. Yan L. Kong H. S.. (2020). Distribution and antifungal susceptibility of candida species causing candidemia in China: An update from the CHIF-NET study. J. Infect. Dis. 221, S139–S147. doi: 10.1093/infdis/jiz573 32176789

[B52] Xiao M. Wang H. Lu J. Chen S. C. Kong F. Ma X. J.. (2014). Three clustered cases of candidemia caused by candida quercitrusa and mycological characteristics of this novel species. J. Clin. Microbiol. 52, 3044–3048. doi: 10.1128/JCM.00246-14 24696025 PMC4136143

[B53] Yadav A. Singh A. Wang Y. Haren M. H. V. Singh A. De Groot T.. (2021). Colonisation and transmission dynamics of candida auris among chronic respiratory diseases patients hospitalised in a chest hospital, Delhi, India: A comparative analysis of whole genome sequencing and microsatellite typing. J. Fungi (Basel) 7, 81. doi: 10.3390/jof7020081 33530297 PMC7910912

[B54] Zhai B. Ola M. Rolling T. Tosini N. L. Joshowitz S. Littmann E. R.. (2020). High-resolution mycobiota analysis reveals dynamic intestinal translocation preceding invasive candidiasis. Nat. Med. 26, 59. doi: 10.1038/s41591-019-0709-7 31907459 PMC7005909

[B55] Zhang L. Xiao M. Arastehfar A. Ilkit M. Zou J. Deng Y.. (2021). Investigation of the emerging nosocomial wickerhamomyces anomalus infections at a Chinese tertiary teaching hospital and a systemic review: Clinical manifestations, risk factors, treatment, outcomes, and anti-fungal susceptibility. Front. Microbiol. 12, 744502. doi: 10.3389/fmicb.2021.744502 34690991 PMC8527005

[B56] Zhang L. Yu S. Y. Chen S. C. Xiao M. Kong F. Wang H.. (2020). Molecular characterization of candida parapsilosis by microsatellite typing and emergence of clonal antifungal drug resistant strains in a multicenter surveillance in China. Front. Microbiol. 11. doi: 10.3389/fmicb.2020.01320 PMC7309193 32612597

